# Transfusion

**Published:** 1875-06

**Authors:** 


### Transfusion.

Prof. Mosler, of Griefswald, has recently reported in the *Berliner Klinische Wochenschrift* (May 17) the details of a case in which a woman who had some hours lain pulseless and unconscious from intestinal hemorrhage due to typhoid fever was immediately and permanently revived by transfusion practiced into her radial artery. Defibrinated blood from a healthy man was used. This is said to be one out of four cases, and the second successful one, in which transfusion has been used in the hemorrhage for enteric fever. There is one point in the case to which we desire to call especial attention. Prof. Mosler performed the operation in the unusual way employed, avowedly to avoid the risk of distending and thereby paralyzing an excessively weak right ventricle. Several cases abroad have proved instantaneously fatal from cardiac paralysis, evidently simply the result of over-distention of a right heart already scarcely able to fulfil its duties. One of the most terrible dramatic scenes we ever saw owed its chief interest to the same misfortune. The patient, a little French boy, in a foreign clime, pale and waxy from advanced leucocythæmia, surrounded by doctors, sat up in bed with an expression of mortal terror as he watched the preparations for the operation; screaming when the trifling incision was made; outwardly calm, but panting, with nostrils distended, as the syringe was introduced into the canula. Suddenly, as the piston went up, a frightful deadly pallor, a look of mortal agony, a start and a cry, with upthrown arms, "Mon Dieu! je vais mourir!" a gasp, a shudder, a heavy fall back upon the pillows, and the life was ended.

In this case, at the autopsy, the cardiac walls were found to be thin, and the muscles degenerated, whilst a large pericardial effusion added to the heart's embarrassment. As there was no reason for suspecting any entrance of air into the veins, the death was evidently wrought out in the manner described.—*Philadelphia Medical Times, Editorial.*

